# Developing a Model for Agile Supply: an Empirical Study from Iranian Pharmaceutical Supply Chain

**Published:** 2013

**Authors:** Ali Rajabzadeh Ghatari, Gholamhossein Mehralian, Forouzandeh Zarenezhad, Hamid Reza Rasekh

**Affiliations:** a *Tarbiat Modares University, Tehran, Iran. *^*b*^*Pharmacoeconomics and Pharma Management*; b *Department, School of Pharmacy, Shahid Beheshti University of Medical Sciences, Tehran,Iran. *; c *Researcher in Institute of Management and Developing of Technology, Affiliated to Tarbiat Modares University, Tehran, Iran*.

**Keywords:** Agility, Pharmaceutical supply chain, Iran

## Abstract

Agility is the fundamental characteristic of a supply chain needed for survival in turbulent markets, where environmental forces create additional uncertainty resulting in higher risk in the supply chain management. In addition, agility helps providing the right product, at the right time to the consumer. The main goal of this research is therefore to promote supplier selection in pharmaceutical industry according to the formative basic factors. Moreover, this paper can configure its supply network to achieve the agile supply chain. The present article analyzes the supply part of supply chain based on SCOR model, used to assess agile supply chains by highlighting their specific characteristics and applicability in providing the active pharmaceutical ingredient (API). This methodology provides an analytical modeling; the model enables potential suppliers to be assessed against the multiple criteria using both quantitative and qualitative measures. In addition, for making priority of critical factors, TOPSIS algorithm has been used as a common technique of MADM model. Finally, several factors such as delivery speed, planning and reorder segmentation, trust development and material quantity adjustment are identified and prioritized as critical factors for being agile in supply of API.

## Introduction

In today’s extremely competition-oriented universal market, productive supply chain management (SCM) has a crucial role and is accepted as a key factor for organizational presentation and competitive advantage (1, 2). The competitive environment needs that companies supply upward quality products and services, deliver quick service response, and improve dynamic capabilities that are in tune with the growing changing business environment (3, 4). Today’s business situation is characterized by an upward level of unpredictability. In this unstable market, firms face aggressive competitive environment due to globalization, technological changes, shorter goods’ life cycles, diminished margins, economic downsized markets and more informed and well-informed customers with unique and quickly changing needs. The focus of supply chain has changed from production efficiency to customer-driven and collaboration synchronization approaches which need a high degree of cooperation among all supply chain partners (5). These changing market situations forces organizations to alter the path their supply chains structured and handled in order to be more responsive to these changes. In order to respond to the challenges and demands of today’s business environment, firms have been undergoing a revolution in terms of implementing novel operations strategies and technologies (6).

 Recent literature in supply chain has addressed this flow and proposes that the key factor to survive in these changing situations is through agility by formation of responsive supply chain (7). In a continuously changing global competitive environment, an organization’s supply chain agility directly affects its ability to produce and give inventive products to its customers in a timely and cost-efficient manner (8). In such an unstable environment, companies require to improve more flexible and robust linkage with partners in order to reply to market situations in a timely manner. Therefore, being agile and capable of quick adjustment to unexpected changes undoubtedly become critical success elements for organizations (9). Furthermore, due to strategic worth, supply chain agility must be operationalized in a way that companies can manage their agility level through their strategic decisions (10). 

The pharmaceutical section plays a significant role in the medical and health system. The pharmaceutical market is heavily regulated in many countries because of the unique nature of demand and supply (11). According to the characteristics of the contest in drug market, governments must balance both clinical and economic interests (12). One of the targets of this supply chain is to assure a continuous flow of drugs to patients at optimal price, with minimal delays, few shortages, and with little room for error (13). A scientific and technological transformation is occurring in the pharmaceutical industry that will make it possible for drug producers to produce profitable new medicines for situations that cannot be treated very well today and for conditions which have formerly persisted against all treatments. Anyway, now several elements are pressing pharmaceutical firms to change their old manners of conducting business. One of these elements is the supply chain which is changing to a source of competitive advantage (14). Finally, the purpose of this paper is to address this question: *“Which critical factors should be taken* *into account by pharmaceutical companies to* *develop an agile model in supplier section?”*

To answer the question, this article benefits from the fuzzy TOPSIS to quantify critical factors. The remainder of the paper is organized as follows: presenting the literature on SCM and a review of pharmaceutical industry, studying the design and basic factors, presenting the results and analysis, discussion, conclusion and implications.

*Literature Review*

*Pharmaceutical industry environment*

The pharmaceutical industry is explained as a system of procedures, operations and organizations involved in the discovery, development and production of medications. The pharmaceutical supply chain (PSC) represents the path through which essential pharmaceutical products are distributed to the end-users at the right quality, at the right place and at the right time (15). The pharmaceutical supply chain is very complicated and greatly responsible to ensure that the appropriate drug, reaches the right people at the right time and in the right situation to fight against sicknesses and sufferings. This is a highly sensitive supply chain that everything less than 100% customer service level is unacceptable as it directly influences the health and safety. The solution that a lot of pharmaceutical industries adopt is to bear a vast inventory in the supply chain to ensure close to 100% fill rate. However, it is a great war to ensure 100% product availability at an optimum cost unless the supply chain processes are streamlined towards customer requirements and demands (16).

The time to market, R and D productivity (Innovations), drugs’ life cycle reduction, government regulations, decreasing exclusive patent life, production flexibility, and increasing cost are the main problems that pharmaceutical industries are facing today. A manufacturer who can adjust the improvement time by 19% can save up to $100 million. At the time of a drug getting delayed to access the market, firm may get rid of around $1 million a day, therefore, the access time to market is so important for pharmaceutical companies in order to gain market share (16). The pharmaceutical market is heavily regulated in many countries because of the unique nature of demand and supply for drugs (17). In accordance with the feature of the competition in drug market, governments must balance both clinical and economic interests (12). Finally, the pharmaceutical section plays a key function in the medical and health system. Characterized with its size of total and aging population, quickly increasing economy and increasing prevalence of chronic diseases (like cardiovascular disease, cancer, and chronic respiratory disease) pharmaceutical industry growth has been increased at a very fast rate (18).

*Pharmaceutical companies in Iran*

On the eve of the 1979 revolution, numerous domestic, foreign, and domestic-foreign private companies were active in Iran›s pharmaceutical sector. By that time, the country›s pharmaceutical sector had been transformed into a market that boasted a $300 million annual cash flow. There were nearly 4,000 kinds of pharmaceutical products available in Iran, 70% of which was provided by imports and the remaining 30% was produced domestically (19). More than half of the latter market served the sales of products under the concession of foreign companies (20). At present, more than 95% of the drug consumption is produced by domestic pharmaceutical companies (18, 21, 22).

*Pharmaceutical supply chain components*

The pharmaceutical supply chain (PSC) like the other industries begins with the sourcing of active and inactive ingredients for approved products. Dosages are planned and packed into different configurations. Products moved along to company’s warehouses, wholesale distributors, retail pharmacies, medicinal organizations (hospital pharmacy), and finally to end-users. The data flow and funds flow start from end customer to producer through different channels (16).

**Figure 1:**
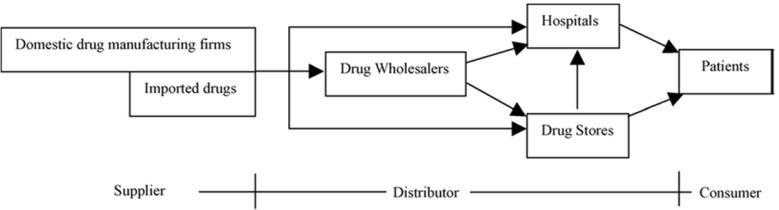
Pharmaceutical supply chain (15

 A supply chain is the arrangement of organizations, their facilities, acts, and activities that are involved in manufacturing and giving a product or service. A typical pharmaceutical supply chain consists of the following members: initially manufacturing, secondary producing, market warehouse/distribution centers, wholesalers, retails/hospitals and patients (23). Previously, under a centrally organized economy, the whole pharmaceutical products were distributed byan owned monopoly firm (first-tier wholesaler) to some regional wholesalers (second-tier wholesalers) who would then deliver the products to local wholesalers (third-tier wholesalers) (24). Among pharmaceutical supply chain components, it has been argued that delivery of medicines has substantial effect on customers’ satisfaction (25). Because of the changing economic system; pharmaceutical supply chain has been reformed. Figure 1 exhibits the new pharmaceutical supply chain**. **In this continuum, wholesalers play as a customer role and patients are considered as consumers.

*The agile supply chain* Supply chain agility has received very consideration recently as a way for organizations to reply in a quick manner to change the business environment and improve their customer service levels. In order to comprehend this concept, it is important to first establish the definition of the agile companies. Agility has been proposed as a reply to the high levels of intricacy and uncertainty in advanced markets (26). According to Naylor *et al. *(1999), “agility means applying market knowledge and a vital corporation to exploit profitable opportunities in a rapidly changing market place”. The relation between agility and flexibility is extensively discussed in the literature (7, 8). It has been proposed that the origins of agility lie in flexible manufacturing systems (27, 28).

**Figure 2:**
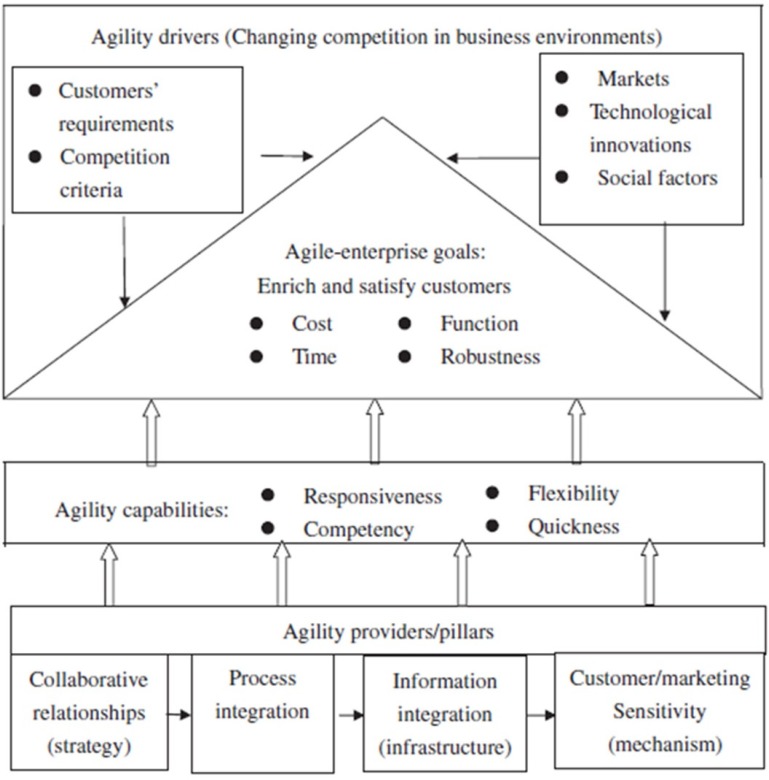
Components of an agile supply chain (29).

The target of an agile enterprise is to enrich/ satisfy customers and employees. A firm basically possesses a set of capabilities for making appropriate replies to changes occurring in its business environment. Anyway, the business statuses in which a lot of companies understand themselves are characterized by volatile and unpredictable demand. Agility might hence, be defined as the ability of a firm to reply rapidly to changes in the market and customer demands. To be really agile, a firm should control a number of differentiating agility-providers. Tseng *et* *al. *(2011) have developed an agile enterprise conceptual model, as shown in Figure 2 (29).

 Hence, these firms need a number of distinguishing attributes to promptly deal with the changes inside their environment. Such attributes include four main elements (30): responsiveness, competency, flexibility/ adaptability and speed. The base for agility is the joining of information technologies, staff, business process organization, innovation and facilities into main competitive attributes. The embracing of agile strategies has some benefits for firms, including quick and efficient reaction to changing market requests; the ability to customize products and services delivered to customers, the capability to manufacture and deliver new products in a cost-efficient mode (8), decreased producing costs, enhanced customer satisfaction, removal of non-valueadded activities and increased competitiveness. Therefore, agility has been advocated as the commerce paradigm of the 21^st^ century and in addition, agility is considered as the winning strategy for becoming a universal leader in an increasingly competitive market of quickly changing customers’ requirements (31, 32).

*Supply chain operations reference (SCOR) model*

In the current study, we will employ some parts of the Supply Chain Operations Reference (SCOR) model according to Supply Chain Council in 2001 (33). The SCOR makes a cross industry structure for the estimation and improvement of supply chain management and execution (34). Five main supply chain processes are captured by the structure of the SCOR model. The processes are planning, sourcing, making, delivering and returning. In part of conceptualization of supply chain agility, it is better to apprise each of them disparately in order to frame the theoretical parts of supply chain agility into a generally accepted business structure (33).

*Agile supplier selection*

In today’s highly competitive environment, enterprises require to take promotion of any opportunity to develop their performance. There has been increasing recognition of the need for a firm to work closely with its supply chain partners in order to optimize its business activities. A key function in the structure formation of any supply chain is that of supply partner selection (35), which is reflected in the increasing research interest in this subject in recent years (36). The agile supply chain (ASC) is a dynamic alliance of member companies, the formation of which is likely to require changing frequently in response of fast-changing markets (37, 38). More recently, in an era of intensified outsourcing, Huang *et al. *(2004) have insisted on the idea of the virtual firm as an effective and viable solution to the problem of fulfilling requirements in a universal market. In ASCs, companies must align with their supply partners to streamline their operations, as well as working with each other to reach the necessary levels of agility throughout the entire supply chain and not just among an individual company. The growing importance of ASCs has concentrated more attention on supply partner selection. In ASC, decision-making about partner selection is particularly challenging, due to the complexity of putting together a network under dynamic conditions. Researchers have generally proposed that the problem of supplier selection under aforementioned conditions cannot be solved effectively and efficiently unless it is separated into several sub-problems, each of which can then be discussed and solved individually (39, 36). For instance, Lorange *et al. *(1992) developed a two-stage supply partner selection approach: first, evaluating the level of compatibility with a candidate partner and then examine the market potential, key competitors and simulating worst-case scenarios after the formation of the partnership. De Boer *et al*. (2001) described the supply chain partner selection process as three important stages, comprising the ‘‘criteria formulation’’ and ‘‘qualification’’ stages in which appropriate partners are identified, followed by the ‘‘choice’’ stage in which a final selection is made from appropriately qualified partners. Che (2010) also developed a two-phase model. In phase 1, suppliers are clustered in accordance to their characteristics for meeting customer needs on multiple agents of cost, quality and time. In phase 2, a multi-criteria optimization mathematical model was constructed on the basis of these clusters (40-42).

The authors could find no studies on the agility of pharmaceutical supply chain in any of the developing countries. This research will contribute to reduce the current lack of aforementioned studies and also it extends agility scale as a key component of PSC into developing countries and into a new sector.

*Fuzzy TOPSIS*

TOPSIS (technique for ordering preference by similarity to ideal solution) technique of solving the multi-criteria decision choosing tasks that implies full and complete information on criteria, was expressed in a numerical form. The method is very useful for solving the real problems; it provides us with the optimal solution or the alternative›s ranking. In addition to this, it is not so complicated for the managers as some other methods which demand additional knowledge. TOPSIS technique would search among the given alternatives and find the one that would be closest to the ideal solution but farthest from the anti-ideal solution at the same time. Modification of the method aims to set a different manner of determining the ideal and anti-ideal point through standardization of linguistic attributes› quantification and introduction of fuzzy numbers in description of the attributes for the criteria expresses by linguistic variables (43).

*Study design*

In this section, we provided a methodology for operationalizing the variables and factors, acquiring the data and determining the reliability of factor grouping. The data used in this study was gathered from questionnaires distributed to the managers of Iranian pharmaceutical companies. The pharmaceutical industry is chosen as it has a heavy and complete supply chain. These types of firms have tried to improve their supply chain performance due to the increasing concerns and importance of supply issues and also manufacturers are seeking methods to improve their performance.

This research is based on supply chain operations reference model (SCOR), and our scope in this paper emphasizes on the supply of API. The questionnaire was designed based on ten critical factors listed in Table 1, which was created in previous studies (44, 45, 29), with 25 questions measuring attitudes including: the chosen response can be strongly disagree, disagree, no opinion, agree, or strongly agree. 

**Table 1:** Agile supply factors.

**Factors**	**Factor dimension**	**Researches**
Planning and reorder segmentation	- Market research and monitoring - Forecast of alternatives Suppliers	Baramichai *et al.*, 2007 (44); Agarwal *et al.*, 2007 (45); Tseng *et* *al.*, 2011 (29); Lin *et al.*, 2006 (46); Swafford *et al*., 2008 (47)
Assessment and prioritizing of suppliers for purchasing	- Quality/cost standards for supplier selection - Maintaining list of prequalified suppliers	Baramichai *et al.*, 2007 (44)
Utilizing of IT tools (UIT)	- E-commerce - Electronic biding - RFID (Radio frequency identification)	Baramichai *et al.*, 2007 (44); Gunasekaran *et al.*, 2008 (6); Agarwal *et al.*, 2007 (45); Swafford, 2003 (10)
Suppliers empowerment	- Operational information sharing - Flexible contract - Partnership with suppliers	Baramichai *et al*. ,2007 (44); Tseng *et al*., 2011 (29); Lin *et al.*, 2006 (46)
Material quantity adjustment (for different orders)	- Order consolidation - Variety of suppliers	Baramichai *et al.*, 2007 (44)
Process integration & performance management	- Co-managed inventory - Collaborative product design& development - Synchronous supply	Agarwal *et al.*, 2007 (45); Christopher, 2000 (7)
Cost reduction(CR)	- Sourcing cost - Inventory cost	Qureshi *et al*., 2008 (48); Agarwal *et al*., 2007 (45); Lin *et al.*, 2006 (46); Swafford, 2003 (10)
Delivery speed (DS) Trust development (TD) Environmental pressure	- Responsiveness rate - Reliability delivery - Trust-based relations with suppliers - Minimizing uncertainty (MU) - Political factor - Economic factors - Social factors	Agarwal *et a*l., 2007 (45); Tseng *et al*., 2011 (29); Sharifi *et al*., 1999 (60) Agarwal *et al*., 2007 (45); Tseng *et al*., 2011 (29); Sharifi *et al.*, 1999 (60) Agarwal *et al.*, 2007 (45); Tseng *et al.*, 2011 (29); Handfield *et* *al.*, 2002 (49) Sharifi *et al.*, 1999 (60); Tseng *et al.*, 2011 (29)

In addition to the above questions, the information related to the basic profile of the interviewees was requested at the end of the questionnaire. The main sampling targets were senior managers, different department’s managers and personnel who were involved in decision making. 

**Figure 3:**
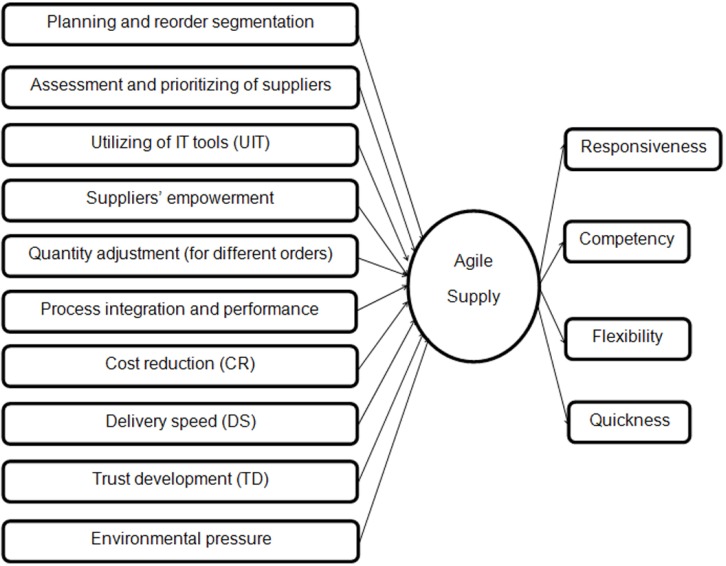
Research model

Our research model is presented in Figure 3. The key dependent variable of interest is agility in supply of API that is expected to be influenced by some independent variables. 

These variables have some subfactors which are shown in Table 1, and as a result, agility can improve responsiveness, quickness, flexibility and competency of suppliers.

*Capabilities of agility*

Agile enterprises require a number of distinguishing capabilities or ‘‘fitness’’ to deal with the change, uncertainty and unpredictability within their business environment. These capabilities consist of four principle elements (50, 51): (1) responsiveness which is the ability to identify changes and respond quickly to them, reactively or proactively, and recover from them; (2) competency which is the ability to efficiently and effectively reach enterprises’ aims and goals; (3) flexibility/adaptability which is the ability to process different processes and achieve different goals with the same facilities; and (4) quickness/speed which is the ability to carry out activity in the shortest possible time. Furthermore, underpinning these four principles is a methodology to integrate them into a coordinated, interdependent system, and to translate them into strategic competitive capabilities (30). These must be taken into account if an organization is to carry out agile enterprise (29).

*Reliability and validity of the questionnaire*

The internal consistency of a set of measurement items refers to the degree to which items in the set are homogeneous. Internal consistency can be estimated using reliability coefficient such as cronbach’s alpha (52). In this research, cronbach’s alpha was calculated 0.9. Content validity is not evaluated numerically; it is subjectively judged by the researchers (53). It is important since the measurement items were based on an extensive review of the literature on various SCM approaches. To gauge the acceptance of the questionnaire, 10 people who were qualified in the field of SCM, participated in a pilot test. The participants suggested adding and omitting some parts of questionnaire. Finally, all the pretest participants expressed strong agreement with the suitability of the questionnaire. The questionnaire was considered finalized after modifying some questions and then, became ready to be delivered. 

*Data collection*

 Data for this study has been gathered using questionnaire that was distributed to 21 pharmaceutical firms which affiliated to three large holding companies. In order to understand the viewpoints on agile supply from key sectors of the pharmaceutical industry, questionnaires were sent to the marketing, sales, information technology (IT), finance, research and development (R & D) and quality assurance and control departments. Accordingly, we chose respondents from managers who had comprehensive knowledge about company’s process, products and general pharmaceutical related issues. The number of questionnaires sent out was 145 and the number of returned ones was 93; a return rate of 64.14 percent. All of the returned questionnaires were complete. Finally, 20% of respondents were top managers and 80% were middle managers according to Table 2.

**Table 2:** Demographics of the respondents

**Position**	**Frequency**	**Percent**
Deputy managing directory	8	8.6
Responsible of production	**9**	9.7
Financial manager	**23**	24.7
Manufacturing manager	**10**	10.7
Quality assurance and control manager	**14**	15.1
Marketing and sales manager	**11**	11.8
Strategic planning manager	**18**	19.4
Total	**93**	100.0

## Results

Data analysis has been done by statistical analysis and also Multiple Attribute Decision Making (MADM) algorithm. In statistical analysis, we have used Student t-tests (one sample t-test), Pearson correlation, and for MADM algorithm, we applied fuzzy TOPSIS technique to prioritize the SCM agility factors. There are many applications of fuzzy TOPSIS in the literature. Chen *et al. *(2006) presented a fuzzy TOPSIS approach to deal with the supplier selection problem in a supply chain system. The TOPSIS method was firstly proposed by Hwang and Yoon in 1981. The basic concept of this method is that the chosen alternative should have the shortest distance from the positive ideal solution and the farthest distance from a negative ideal solution. A positive ideal solution is a solution that maximizes the benefit criteria and minimizes cost criteria (54, 55, 43); whereas, a negative ideal solution maximizes the cost criteria and minimizes the benefit criteria. In the classical TOPSIS method, the weights of the criteria and the ratings of alternatives are known precisely and the crisp values are used in the evaluation process. However, under many conditions, crisp data are inadequate to model real-life decision problems. Therefore, the fuzzy TOPSIS method is proposed, in which the weights of criteria and ratings of alternatives are evaluated by linguistic variables represented by fuzzy numbers to deal with the deficiency in the traditional TOPSIS (56).

*T-test analysis*

In the first step, we have done t-test analysis for determining the situation factors. Table 3 shows the result of t-test and all factors have the significant difference with cut point 3.

**Table 3:** Result of mean difference (one sample t-test).

**Factors**	**T-statistic**	**mean**	**Standard deviation**
**Planning and reorder segmentation**	49.7	3.8	0.96
**Assessment and prioritizing of suppliers for purchasing**	57.5	4.3	0.98
**Utilizing of IT tools (UIT)**	33.5	3.5	0.94
**Suppliers empowerment**	48.0	3.7	0.97
**Material quantity adjustment (for different orders)**	42.7	3.2	0.96
**Process integration and performance management**	41.5	3.1	0.97
**Cost reduction(CR)**	41.9	3.1	0.99
**Delivery speed (DS)**	52.5	4.01	0.99
**Trust development (TD)**	56.9	3.8	0.95
**Environmental pressure**	43.7	3.3	0.97

*Correlation analysis*

We have used Pearson correlation to test the relations among critical factors. It means what›s the inter correlation among factors. The results indicated that these factors have been generally correlated with each other.

*Result of fuzzy TOPSIS*

In order to apply fuzzy TOPSIS, we have converted the language terms to fuzzy numbers

according to Table 4

**Table 4:** Language term.

**Very low**	1	(0,0.1,0.2)
**Low**	2	(0.1,0.25,0.4)
**Medium**	3	(0.3,0.5,0.7)
**High **	4	(0.6,0.75,0.9)
**Very high**	5	(0.8,0.9,1)

 As shown in Table 5, the priorities of basic factors according to fuzzy TOPSIS’s results show that the delivery speed (DS) has the first priority and planning and reorder segmentation, trust development (TD), material quantity adjustment (for different orders), cost minimization (COM), assessment and prioritizing of suppliers for purchasing, environmental pressure, suppliers empowerment, process integration and performance management and finally utilizing IT tools (UIT) are considered.

**Table 5:** Ranking of the main factors.

**Factors**	**Ci+ (rank of TOPSIS)**
Delivery speed (DS)	0.33
Planning and reorder segmentation	0.41
Trust development (TD)	0.45
Material quantity adjustment (for different orders)	0.57
Cost reduction(CR)	0.70
Assessment and prioritizing of suppliers for purchasing	0.81
Environmental pressure	1.26
Suppliers empowerment	1.27
Process integration and performance management	1.39
Utilizing of IT tools (UIT)	1.78

## Discussion and Conclusion

Agility is a key ability in the revolutionary turning of the business environment into a turbulent place of competition and struggle for success. Agility is the ability to detect the changes in the business environment, and respond to them by providing the appropriate capabilities. Strategic intent to become agile and leveraging the core competencies of the company towards achieving the competitive advantage is essential. Every company should understand the circumstances it deals with, the threats it receives from the business environment and the opportunities that would bring them prosperity and success. These concepts have been put together in the form of a methodology that suggests a realistic understanding of the manufacturers’ business environment and some steps that would lead them to resolve the difficulties and problems and also the ways to take advantage of the emerging opportunities. Today, organizations encounter dynamic and changing environments where product life cycles are short and environmental pressures make a lot of uncertainty that lead to more risk management. Organizations need agility to deal with these situations and they should track these categories not only in the organization but also in their entire supply chain (57, 58).

In this study, all attempts aimed at providing an efficient and optimized model for agility of supply chain in the pharmaceutical Industry. To do so, first there is an attempt to identify factors affecting supply chain agility followed by providing the relationship between these factors and supply chain agility capabilities. Ten main indicators and 24 sub-indices were identified as the most important factors affecting the process of supply agility; the main indicators include planning and reorder segmentation, assessment and prioritizing of suppliers for purchasing, utilizing of IT tools (UIT), suppliers empowerment, material quantity adjustment (for different orders), process integration and performance management, cost reduction (CR), delivery speed (DS), trust development (TD) and environmental pressure. However, among these 10 factors, the index of product›s delivery speed was identified as the first rank which represented the degree of importance of this indicator in the agility of the API supply. Speed of delivery refers to the ability of products delivery faster than competitors (59). According to Sharifi *et al. *(1999), this indicator along with sub-indicators (high rate of response to orders and reliability of delivery) will directly increase the speed of supply chain. The second indicator is planning and reordering the segmentation which includes coordinated and collective efforts among supply chain partners in order to achieve the same objectives (60, 61). These objectives include achieving a supply chain system with maximum efficiency and optimum profitability.

Therefore, many researchers (45, 26, 62) noticed that the planning and reorder segmentation will increase accountability and flexibility of the supply chain. The third important and influential parameter that affects supply›s agility is trust development. This indicator along with subindicators of mutual trust between supplier and manufacturer can also influence directly on competency and accountability of the supply chain. The fourth indicator includes material quantity adjustment (for different orders) which will promote accountability and flexibility of chain supply by notifying suppliers about exact amount of demand and scope of materials according to Sharifi *et al. *(1999) (60). Cost minimization beside sub-indicators of the cost of sourcing and increased cost resulting from excess inventory in the warehouse is considered as the fifth factor affecting the agility of supply process in this model. Certainly each business wants to reduce the costs since it has many positive effects. In the agile supply chain, we are looking for reducing the costs both inside and outside the organization that directly or indirectly impacts on the finished product›s cost (63). According to Agarwal *et al. *(2007), cost reduction can promote accountability of supply chain as well (45).

The sixth factor is the assessment and prioritizing of suppliers for purchasing. Based on the study of Baramichai *et al. *(2007), it is considered as a key factor affecting the agility of supply process. In connection with the seventh factor, Sharifi *et al. *(1999) addressed the change in consumer demands as the most important environmental pressure, and in addition to the aforementioned issue, Lin *et al. *(2011) stated that social factors should be considered as an effective environmental pressures in an agile supply chain. The eighth effective index is supplier’s empowerment that is related to three sub-indices of transferring of ideas about product features, taking advantage of flexible contracts in relation with the product characteristics and relationship based on collaboration (44, 60, 29). According to Sharifi *et al. *(1999), they can directly increase the eligibility and flexibility of the supply chain (60). Process integration and performance management is the ninth effective index on agility of the supply chain. Process integration stands for the collaboration between buyers and suppliers, collaborative product development and public systems for information sharing (7). Sharifi *et al. *(1999) noticed that the integration of processes along with sub-indices of coordinated management of inventory, cooperation in product design and the simultaneous supply can directly increase the eligibility and flexibility. In the obtained model of supply›s agility, the last identified effective factor is utilizing the IT tools (UIT) while, Breu *et al. *(2001) stated that information systems are integral parts of agile supply chain and they will increase the speed and flexibility of it (60, 64).

*Managerial Implications*

During the recent decades, SCM has become a popular agenda for both the pharmaceutical industry and non-pharmaceutical industries. These pharmaceutical companies can successfully minimize and manage the risk and uncertainty inherent in their supply chain value stream. Globalization, outsourcing, single sourcing, just-in-time supply chain management, lean and agile supply chain have made pharmaceutical supply chain more sensitive to environment. Accordingly, to survive and thrive in the 21^th^ century economy, pharmaceutical companies should learn how to encounter ongoing challenges in their environment. This forces pharmaceutical firms to select a new way of operating that gives them ability to be flexible and respond quickly to unpredictable changes. So, to succeed, pharmaceutical firms must consider supply chain management deeply, in order to become resilient to unexpected disruptions in their supply chain. Finally, it should be said that due to the unbelievable relationships between the response to consumer’s requirements and firm’s success (like profitability and corporate social responsibility), pharmaceutical firms are supposed to extensively pay attention to their supply chain activities.
